# ENHANCING THE AGING WORKFORCE: THE CASE FOR ACCREDITATION OF GERONTOLOGY PROGRAMS

**DOI:** 10.1093/geroni/igae098.0819

**Published:** 2024-12-31

**Authors:** Maria Henke

**Affiliations:** University of Southern California, Los Angeles, California, United States

## Abstract

For over five decades, gerontology programs have been attempting to address the needs of aging populations by preparing professionals equipped with specific knowledge, skills, and competencies. Of paramount concern to any gerontology program is that their graduates are prepared to be valuable members of the workforce. This can be done by establishing cohesive standards within the field so that future employers recognize and value the qualifications of gerontology graduates. These standards can be established and improved upon by creating a dialogue with future employers to more fully understand their workforce needs as well as to clearly communicate the value graduates from accredited gerontology programs bring to their organizations. It is important that there is a core of consistency from program to program. This consistency ensures that regardless of the specific focus or emphasis of each program, employers will recognize the particular skill set a gerontologist brings to their organizations and businesses. The Academy for Gerontology in Higher Education (AGHE) is the educational arm of the Gerontological Society of America (GSA). AGHE has formulated a set of core competencies for gerontology programs. These competencies serve as the cornerstone of the accreditation process facilitated by Accreditation for Gerontology Education Council (AGEC), providing programs with a structured pathway towards recognition and validation. With the framework for accreditation established, it is imperative for programs to embark on this journey, ensuring alignment with the rigorous standards and expectations set forth by the field.

